# *Plasmodium falciparum* clearance time in Malawian children with cerebral malaria: a retrospective cohort study

**DOI:** 10.1186/s12936-021-03947-0

**Published:** 2021-10-18

**Authors:** Alexuse M. Saidi, Geoffrey Guenther, Rima Izem, Xiaojun Chen, Karl Seydel, Douglas Postels

**Affiliations:** 1 Blantyre Malaria Project, University of Malawi College of Medicine, Blantyre, Malawi; 2 Department of Pediatrics, Children’s National Medical Center, Washington, DC USA; 3 Division of Biostatistics and Study Methodology, Children’s National Research Institute, Washington, DC USA; 4 Department of Epidemiology, The George Washington University School of Public Health, Washington, DC USA; 5 Department of Biostatistics and Bioinformatics, The George Washington University, Washington, DC USA; 6 Department of Osteopathic Medical Specialties, College of Osteopathic Medicine, Michigan State University, East Lansing, MI USA; 7 Division of Neurology, The George Washington University/Children’s National Medical Center, Washington, DC USA; 8 Present Address: Statistical Methods and Consulting, Novartis, Basel, Switzerland

**Keywords:** Malawi, Cerebral malaria, Paediatric, Artesunate, Quinine, Clearance time

## Abstract

**Background:**

Standard treatment for both uncomplicated and severe malaria is artemisinin derivatives. Delayed parasite clearance times preceded the appearance of artemisinin treatment failures in Southeast Asia. Most worldwide malaria cases are in sub-Saharan Africa (SSA), where clinically significant artemisinin resistance or treatment failure has not yet been detected. The recent emergence of a resistance-conferring genetic mutation in the *Plasmodium falciparum* parasite in Africa warrants continued monitoring throughout the continent.

**Methods:**

An analysis was performed on data from a retrospective cohort study of Malawian children with cerebral malaria admitted between 2010 and 2019 to a public referral hospital, ascertaining parasite clearance times across years. Data were collected from patients treated for severe malaria with quinine or artesunate, an artemisinin derivative. Parasite density was determined at admission and every subsequent 6 h until parasitaemia was below 1000 parasites/µl.The mean parasite clearance time in all children admitted in any one year was compared to the parasite clearance time in 2014, the first year of artesunate use in Malawi.

**Results:**

The median population parasite clearance time was slower from 2010 to 2013 (quinine-treated patients) compared to 2014, the first year of artesunate use in Malawi (30 h (95% CI: 30–30) vs 18 h (95% CI: 18–24)). After adjustment for admission parasite count, there was no statistically significant difference in the median population parasite clearance time when comparing 2014 with any subsequent year.

**Conclusion:**

Malaria parasite clearance times in Malawian children with cerebral malaria remained constant between 2014 and 2019, arguing against evolving artemisinin resistance in parasites in this region.

**Supplementary Information:**

The online version contains supplementary material available at 10.1186/s12936-021-03947-0.

## Background

Malaria continues to be a leading cause of hospital admission and mortality in children across sub-Saharan Africa (SSA) [1]. In 2019 alone, there were 229 million malaria cases in 87 malaria-endemic countries. Ninety-four per cent of the total reported cases and deaths were from Africa. Globally there were 409,000 deaths, the majority in children under the age of five years [1]. Of the five parasite species that infect humans, *Plasmodium falciparum*, highly prevalent in Africa, is the most common cause of severe illness.

The current first-line therapy for uncomplicated malaria is a combination of at least two anti-malarial medications. A derivative of artemisinin is combined with a second longer-acting drug, a technique known as artemisinin-based combination therapy (ACT) [2]. Monotherapy for uncomplicated malaria treatment is discouraged as it may lead to the emergence of drug resistance [2].

The global standard first-line therapy for severe malaria syndromes: malaria infection with end-organ dysfunction, is intravenous artesunate, an artemisinin derivative. Compared to quinine, artesunate decreases mortality without an increase in the rate of neurological morbidity in survivors, in both adults and children [3, 4]. Artesunate is safer, more easily administered and clears malaria parasites more rapidly than quinine. It is unclear whether more rapid parasite clearance is causally associated with decreased mortality risk.

The superior clinical outcomes of artemisinin derivatives are being threatened by the emergence of drug resistance. Resistance, usually preceded by delays in parasite clearance time after treatment initiation, has already appeared in the Greater Mekong Sub-Region [4]. Delayed clearance is defined as an increase in the time it takes to clear *P. falciparum* from the peripheral blood after administration of an appropriate dose of anti-malarials [5]. Molecular resistance markers have since been detected in population-based surveys in SSA [6, 7] but no clinical resistance to artemisinins, including artesunate, has yet been observed.

Ongoing assessment of delayed parasite clearance times is challenged by the heterogeneity of clinical care, laboratory collection times and parasite density measurements in many clinical studies. Detected changes in parasite clearance times, if found, may be due solely to comparisons being made between patient populations that vary across any of these factors. If changes in parasite clearance are to be detected, minimizing variability in the patient population under assessment and their clinical care will decrease error. Using data previously collected from a long-standing study of cerebral malaria pathogenesis [8], parasite clearance times in children with cerebral malaria were assessed. Other than the change from quinine to artesunate in 2014, enrolment criteria and clinical care of all enrollees, including anti-pyretic, antibiotic and anticonvulsant use, was constant across years. The goal was to determine parasite clearance times annually and compare them through time, to assess whether changes had occurred prior to and after the introduction of artesunate in 2014.

## Methods

Included patients were admitted to a paediatric research unit specialized in the care of children with cerebral malaria between 2010 and 2019. The parent study, located at Queen Elizabeth Central Hospital in Blantyre, Malawi, enrolled children 6 months to 14 years old with World Health Organization-defined cerebral malaria (Blantyre coma score of ≤ 2, *P. falciparum* parasitaemia on peripheral blood smear, and no other known cause of coma) [9]. Informed consent was provided by a child’s parent or guardian prior to enrolment. Ethical review of the parent study was performed by the University of Malawi College of Medicine Research Ethics Committee and Michigan State University (USA). At the time of consent, parents agreed to the secondary analysis of de-identified data, which were used in these analyses.

Children admitted between 2010 and 2013 were treated with quinine 20 mg per kg intravenously, infused over 4 h, followed by 10 mg per kg every 8 h, infused over 2 h. Children enrolled between 2014 and 2019 received artesunate intravenously at admission and every 12 h afterwards for a minimum of 3 doses according to the Malawi Ministry of Health guidelines in effect at the time. From 2014 to 2016, artesunate dosing was 2.4 mg per kg for all patients, and from 2017 onward the dose was increased to 3.0 mg per kg for children weighing less than 20 kg. After completion of artesunate therapy, children received a full course of enteral artemether-lumefantrine, administered by nasogastric tube if the participant was unable to swallow.

Thick and thin blood films were collected at admission and every 6 h until two consecutive parasite counts were negative (no parasites viewed when searching 100 high-power fields). Blood thick films were fixed with low heat for one minute, dipped in a filtered Field stain A for one minute, washed in distilled water, dipped into Field stain B for 30 s, washed again in a separate distilled water container, and air dried. Thin films were first air dried, fixed in absolute methanol for 30 s, air dried again, dipped into Field stain B, washed in distilled water, dipped into Field stain A, washed again, and air dried.

Peripheral parasite density was determined by counting the number of parasites against 500 white blood cells (WBCs) with two tally counters on the thick smear under the light microscope using a 100X magnification lens. Counting was switched to a corresponding thin film if 100 parasites or more were observed in every field. On the thin film, infected red blood cells (iRBCs) were counted in a total of 500 RBCs. Parasitaemia was calculated using either RBC counts for thin films or WBC counts for thick smears determined on admission and analysed on a Coulter A^C^.T5diff AL (Beckman Coulter Life Sciences, Indianapolis, Indiana, USA). Microscopists were tested annually using a malaria slide quality assurance test set from Central Laboratory Services (Johannesburg, South Africa).

Plasma for histidine rich protein 2 (HRP-2) concentrations were collected at admission and archived for later analysis. Admission quantitative HRP-2 was determined after hospital discharge from samples frozen at – 80 ℃. The manufacturer’s protocol was used with the modification of incubations being performed at 37 ℃ (Cellabs, Brookvale, Australia). The plate was analysed using an ELx800 reader at 450 nm (BioTek Instruments, Winooski, Vermont, USA). Plasma HRP-2 concentrations were calculated by comparing the results from patient samples with a standard curve generated from analysis of a recombinant stock. All results that fell outside the linear range were re-analysed after appropriate dilution.

### Statistical analysis

Admission demographic, laboratory and outcome characteristics between patients treated with quinine and artesunate were compared. Summaries of these data included means and standard deviations for continuous variables or counts and frequencies for categorical variables. Characteristics between quinine-treated and artesunate-treated patients were compared using t-tests for continuous characteristics, or Chi square tests for categorical variables. Peripheral parasite densities and HRP-2 levels were logarithmically transformed to stabilize the variance when making comparisons.

To evaluate subject-level and cohort-level parasite clearances, graphical representations of parasite count (natural logarithm scale, y-axis) over time in hours post-admission were created. Linear interpolation to subject-level curves from discrete measures, and boxplot of parasite measures at each measurement time, were used to establish cohort-level summaries. Based on previous work on parasite clearance dynamics, parasite clearance time was defined as the time (in hours) between admission and the first post-admission parasite count of less than 1000 parasites per µl [10]. Patients with fewer than 1000 parasites/µl on admission were excluded from all analyses. Subjects who did not reach a parasite count of less than 1000/µl during hospitalization were censored at the last observed measurement.

Two time-to-event analyses were performed. Unadjusted analyses comparing time-to-event curves over years included Kaplan–Meier curves of time to the first parasite count less than 1000 parasites/µl by anti-malarial administered, and by year within quinine-treated and artesunate-treated patients. A log-rank test was used to compare time-to-event curves by anti-malarial (quinine *vs* artesunate), and across years within quinine-treated and artesunate-treated children.

Cox regression analysis was used to compare time to < 1000 parasites/µl in quinine- and artesunate-treated patients, and to compare enrolled participants across calendar years after adjustment for admission parasite count. A model-based p-value (Wald test) and 95% confidence interval using these adjusted analyses was calculated. In all results, a p value less than 0.05 was considered to show a statistically significant difference between groups. All analyses were performed using the R software package version 4.0.2 (R Foundation for Statistical Computing; Vienna, Austria).

## Results

**Fig. 1:**
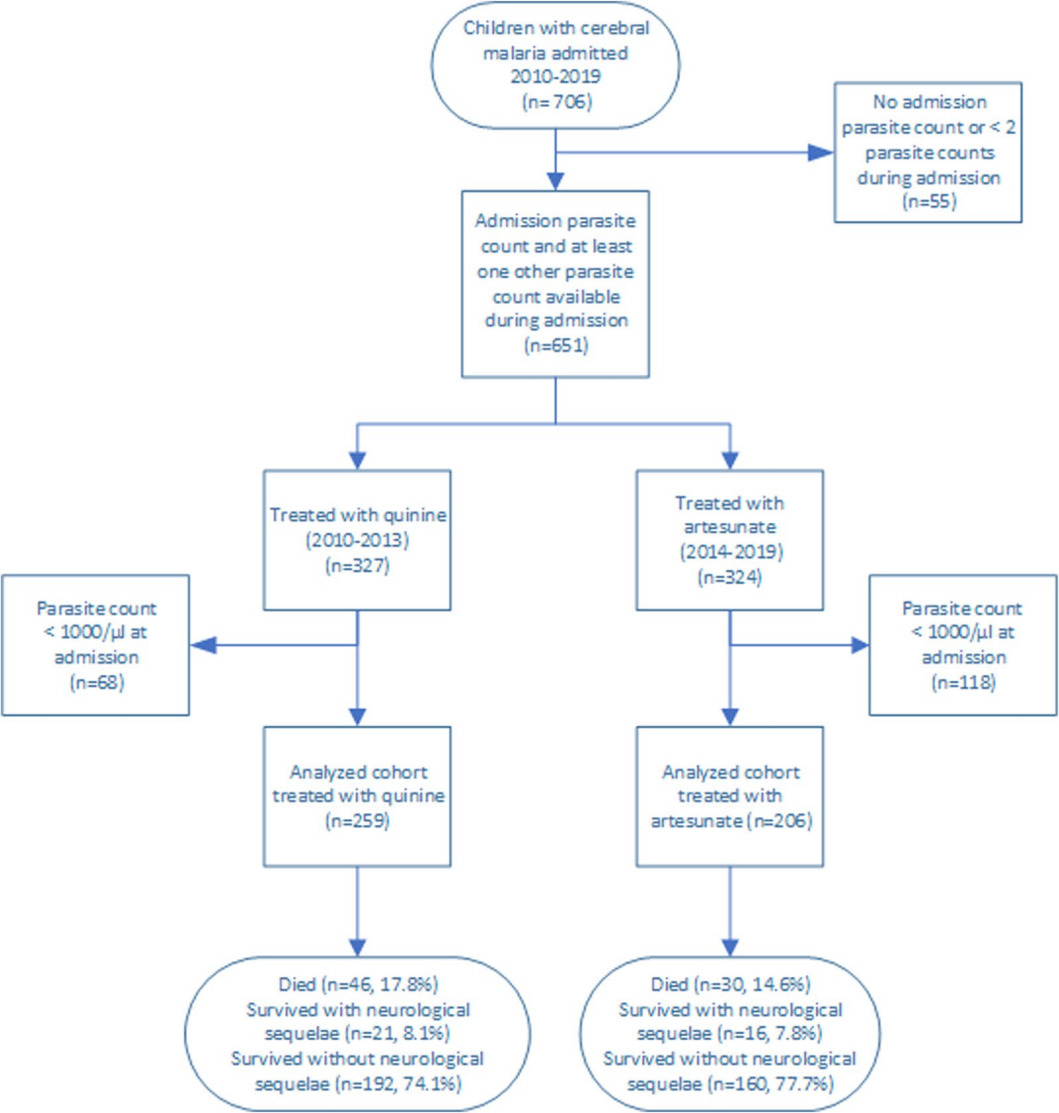
Flow chart for patient recruitment

Between January 2010 and June 2019, 706 children with cerebral malaria were enrolled in the parent study (Fig. 1). Fifty-five children were excluded as they had either no admission parasite density or fewer than two parasitaemia measurements during admission. After exclusion of those with < 1000 parasites/µl, 465 children remained, 259 received quinine (admitted 2010–2013) and 206 received artesunate (2014–2019). Those who received quinine were more likely to have had splenomegaly and also had higher admission glucose and higher parasite and HRP-2 levels compared to those who received artesunate (Table 1). There were no statistically significant differences in either admission Blantyre Coma Score (p = 0.177) or outcomes (mortality or neurological morbidity, p = 0.732) when comparing children treated with quinine or artesunate.

**Table 1:** Demographic and clinical characteristics of children with cerebral malaria included in the final analysed population

Demographic, clinical, outcome measure	Anti-malarial		P value for difference
	Quinine-treated (2010–2013) (n = 259)	Artesunate-treated (2014–2019) (n = 206)	
Age (months): mean, SD	50.9 (28.1)	49.9 (29.8)	0.711
Gender: N male (% male)	136 (52.5)	109 (52.9)	1.000
Splenomegaly (%)	33.6	22.3^a^	0.009
Glucose mmoL/L: mean (SD)	7.08 (3.49)	6.14 (2.84)^b^	0.002
Haematocrit (admission) mean (SD)	22.6 (6.45)^c^	23.8 (6.74)	0.052
Parasites/µl: mean (SD)	260,872 (458,810)	187,612 (278,800)	
Log (parasites): mean (SD)	11.48 (1.63)	10.84 (1.94)	< 0.001
HRP-2 (ng/ml): mean (SD)	7,909 (10,028)	5441 (10,693)	
Log (HRP-2): mean (SD)	7.30 (2.65)	6.96 (2.23)	0.001
Pre-treatment with anti-malarial (%)	222 (85.7)	180 (87.4)	0.70
Blantyre Coma Score: N (%)			0.18
0	22 (8.5)	26 (12.6)	
1	103 (39.8)	68 (33.0)	
2	134 (51.7)	112 (54.4)	
Outcomes^d^			0.732
Died: N (%)	46 (17.8)	30 (14.6)	
Neurological sequelae at hospital discharge: N (%)	21 (8.1)	16 (7.8)	
Full recovery: N (%)	188 (72.6)	158 (76.7)	

*HRP-2 *histidine rich protein 2. Variables compared using t-test for continuous variables and Chi-squared tests for categorical variables. P < 0.05 was considered statistically significant

^a^n = 205

^b^n = 204

^c^n = 258

^d^n = 255 in quinine era and 204 in artesunate era

Based on prior published work [10], post-treatment parasite density curves were anticipated to have three characteristics:

1. A lag (time between administration of intravenous anti-malarials and beginning of negative slope of parasite density curve)
2. An exponential decay (linear after logarithmic transformation)
3. A ‘tail’ which included most measurements with parasite densities of less than 1000/µl.

**Fig. 2:**
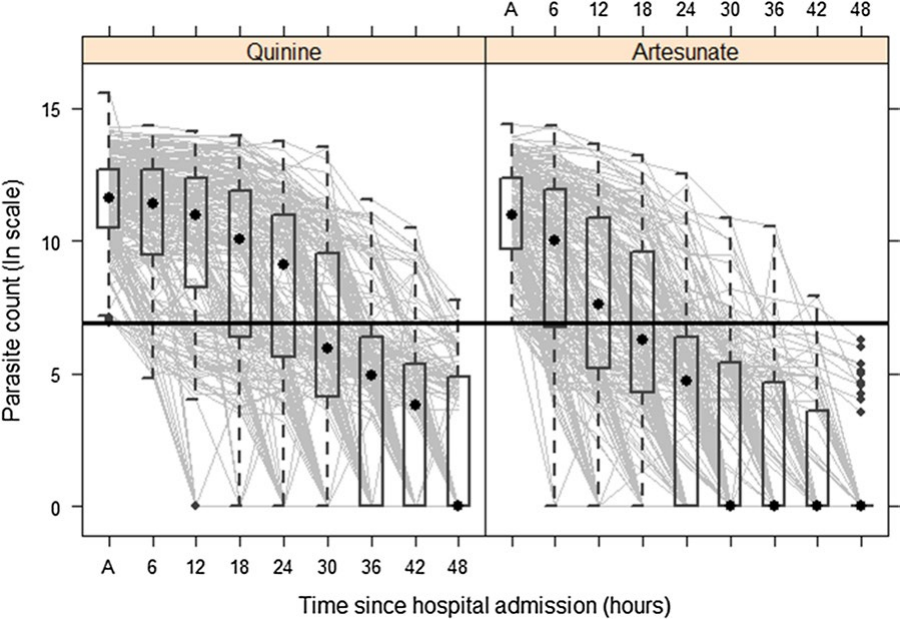
Parasite decay curves for children treated with quinine (left) and artesunate (right) A = admission. The dark horizontal line represents 1000 parasites/µl, the cutoff used in the analysis

On average, these features were noted when plotting population averages through time, for both quinine-treated and artesunate-treated patients (Fig. 2). Qualitatively, children treated with artesunate had a shorter lag time, a steeper decay and a short tail, compared to children treated with quinine. Given the differences in both the lag and decay phases, the choice of a comparative metric such as slope of the decay phase was rejected. Instead, a more integrative metric: time to parasite clearance, was selected. Given the inconsistencies of malaria microscopy at very low parasitaemia levels, a cut-off of 1000 parasites/µl as the endpoint of parasite clearance was chosen rather than absolute zero [11].

**Fig. 3:**
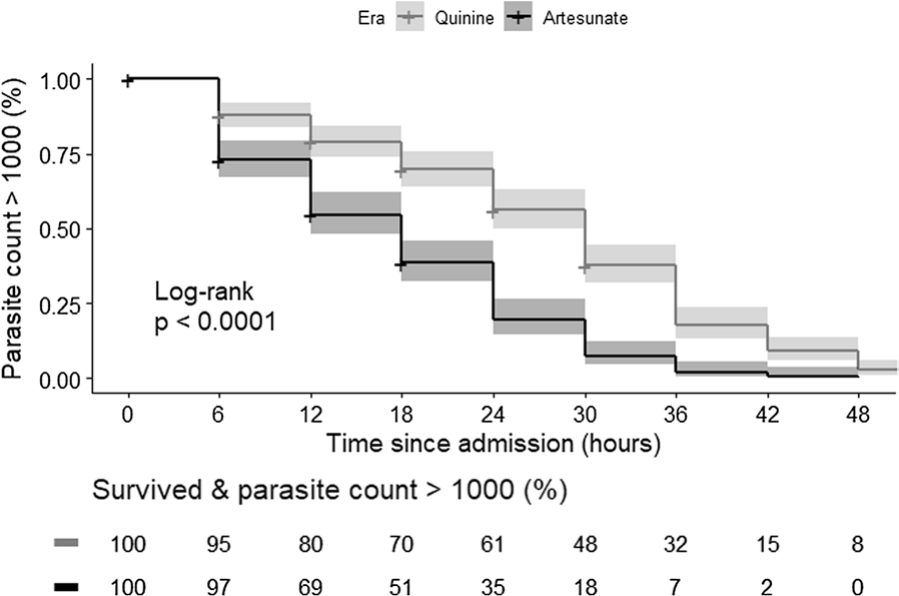
Parasite clearance in quinine- and artesunate-treated patients

Children treated with artesunate achieved a post-treatment parasite count of less than 1000/µl more rapidly than those treated with quinine (log-rank test p-value < 0.05) (Fig. 3). The Kaplan–Meier curves separate as early as 6 h post admission and remain separated over time, with larger difference between the two curves at 24 h and thereafter.

**Fig. 4:**
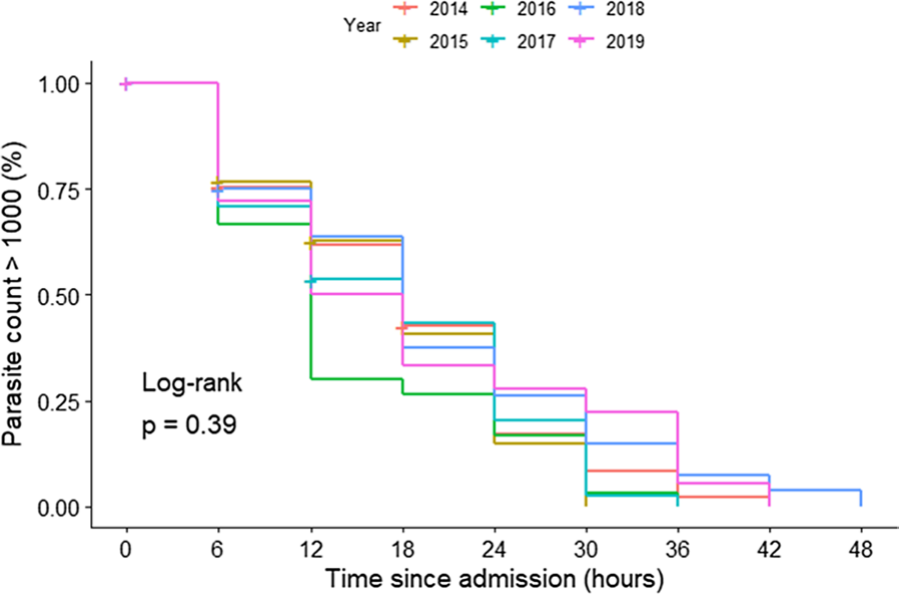
Parasite clearance for artesunate-treated patients with cerebral malaria, 2014–2019 Participant counts are included as Additional file 1: Table S1

After adjustment for admission parasite count, parasite clearance was significantly slower in 2010 to 2013 compared to the first year of introduction of artesunate 2014 (Table 2). Clearance did not significantly change in the years after 2014. Kaplan–Meier curves plotted for the years 2014 and after showed no separation, reflecting no change in clearance rates time across years after 2014 (Fig. 4 and Additional file 1: Table S1).

**Table 2:** Results from Cox proportional hazard model analyses of time to parasite density less than 1000 per µl, adjusting for baseline parasite density, year and anti-malarial administered

	Year	Number of subjects	Hazard ratio (95% CI) *	Hours to parasite reduction to < 1000 parasites/µl median (95% CI)
All quinine years	2010–2013			30 (30,30)
Individual years	2010	118	0.34 (0.24, 0.48)	30 (30,36)
	2011	68	0.46 (0.32, 0.68)	30 (24,30)
	2012	38	0.29 (0.18, 0.47)	24 (18,36)
	2013	35	0.57 (0.36, 0.90)	24 (18,30)
All artesunate years	2014–2019			18 (12,18)
Individual years	2014	53	1.00	18 (18,24)
	2015	30	1.36 (0.86, 2.17)	18 (12,24)
	2016	30	1.20 (0.76, 1.90)	12 (12,18)
	2017	45	1.25 (0.82, 1.89)	18 (12,24)
	2018	29	0.85 (0.53, 1.36)	18 (12,24)
	2019	19	0.95 (0.55, 1.63)	15 (12,36)

^*^All hazard ratio estimates are relative to reference year 2014, the first year of artesunate use in Malawi

## Discussion

Resistance to the artemisinin class of anti-malarials has emerged in recent years as a major threat to the global fight against malaria. The most recent data from Cambodia, Thailand and Vietnam show that first-line ACT medication has a treatment failure rate of approximately 50%, raising serious concerns about future treatment options [11]. Several candidate genes are implicated in artesunate resistance mechanisms, the most consequential of which is the *P. falciparum K13* (*Pfkelch13*) propeller domain, mutations in which are associated with slower parasite clearance times [11].

Development of true artemisinin resistance is typically preceded by progressive delays in parasite clearance times (measured in hours) after anti-malarial therapy is administered. A recent trial enrolling 150 children with severe malaria in Uganda showed no evidence of resistance to artesunate [12]. An ACT treatment failure rate of 19.5% was observed during World Health Organization monitoring in Malawi in 2010 [13], although evidence of resistance has not been borne out in subsequent studies [14, 15]. Indeed, no convincing evidence of clinically significant resistance to artemisinins has yet been found in SSA, although recent in vitro studies have shown de novo development of *Pfkelch13* mutations in Rwanda [16]. Consistent with these studies, the current study shows no increase in parasite clearance time since the introduction of artesunate in 2014. However, the likely future development of artemisinin-resistant *P. falciparum* in Africa is of grave concern to clinicians and malaria researchers as it would have devastating effects on populations living in endemic areas [17].

Ongoing monitoring of parasite clearance times is warranted to alert clinicians and public health officials that true resistance is approaching. Responses might include a temporary return to quinine (possibly in conjunction with other anti-malarials), use of other intravenous severe malaria treatments (in development), changes in artesunate dosing or schedule, or additions of adjunctive therapy to increase artesunate effectiveness. Fortunately, as of today, all these mitigation strategies remain theoretical. Like others, this study showed that compared to quinine, artesunate produces more rapid parasite clearance, measured as the time from admission to a parasite count less than 1000/µl. This appears to be primarily driven by a shorter lag phase and a steeper parasite clearance curve. With artesunate, parasite clearance begins more rapidly once the child is hospitalized and intravenous anti-malarials administered, shortening the time to reach parasite concentrations below which a patient’s symptoms will likely improve.

Previous large studies have shown that populations treated with artesunate have more rapid parasite clearance times and lower mortality rates than populations treated with quinine [3, 4]. This study did not show such a mortality benefit when comparing the two anti-malarials, but the number of participants enrolled was much lower than in these clinical trials. It is unclear whether there is a causal relationship between more rapid clearance and improved clinical outcomes. Future studies with larger sample sizes should consider exploring whether more rapid parasite clearance time is the main driver of artesunate’s reduction in mortality.

This analysis has advantages over those previously conducted. The study population was Malawian children with cerebral malaria, and therefore relatively homogenous. Children were treated on the same inpatient unit and, other than the anti-malarial used, there were no differences in clinical care received or laboratory sample collection and analysis across enrolment years. The catchment area did not change over the period of the study. Previous studies have revealed that parasite clearance times may differ with small changes in geographical location [18]. If delayed parasite clearance times across years are to be detected, it is advantageous to compare patients with a similar disease phenotype, clinical care and geographic locale across time.

It is possible that delayed clearance time could appear in only some children with specific severe malaria syndromes or even cases of uncomplicated malaria. This study, by limiting enrolments to children with cerebral malaria, would not detect this potentiality. Many children enrolled were pre-treated with anti-malarials prior to hospital presentation. This likely decreased their admission peripheral parasite concentrations and caused some to be excluded from these analyses due to parasitaemias below the 1000/µl threshold. Studies that assess parasite clearance through the entire clinical course (from initial symptoms until outcome) would be advantageous to better characterize early changes in parasite-host dynamics.

This study has several limitations. Pre-hospital anti-malarial therapy was not well documented, likely resulting in patients presenting to the hospital in different phases of anti-malarial treatment. Heterogeneity of pre-admission treatment type and duration may have resulted in comparable heterogeneity in the phase of parasite clearance. Clearance rates may be further confounded by differing susceptibilities of malaria parasites to anti-malarial medications depending on their life cycle stage [19]. Patients may present for care with differing parasite stage compositions. It is possible that this would lead to different initial susceptibilities to either quinine or artesunate. Population level analysis will mask these differences. In addition, using decrease in parasitaemia to 1000 parasites/µl as a metric, avoids the resulting differences in the length of the tail portion of the clearance curve as a confounder, as the tail phase begins at parasitaemia levels below this cut-off.

It is possible that immunity or age may influence rates of parasite clearance. Previous studies have not revealed an association between age and parasite clearance time [20, 21]. In this analysis, participants treated with quinine or artesunate were similar in age, decreasing the likelihood that age-dependent immunity varied across years or treatments received.

Microscopy, which is dependent to operator skill and experience, is insensitive to low levels of parasitaemia. The use of a parasitaemia cut-off of 1000 parasites/µl may decrease the likelihood of some operator-dependent variability as this level of parasitaemia is more readily detectable by most microscopists. The use of microscopy to determine levels of parasitaemia may be phased out of practice in the future as newer detection techniques with lower thresholds (e.g., polymerase chain reaction (PCR)) have been developed, although these newer techniques remain less widely available. Levels of parasitaemia below the limits of detection by microscopy remain infectious to mosquitoes and are likely to be a driver of disease transmission [22]. Very low levels of parasitaemia are less likely to have relevance to studies of pathogenesis, however. Continued use of less sensitive but more widely available methods of parasite quantitation remain suitable for pathogenesis, epidemiological monitoring and drug efficacy trials.

## Conclusions

For Malawian children with cerebral malaria, there was no evidence of delayed malaria parasite clearance time since the introduction of the use of artesunate in 2014. Although clinicians and public health officials in SSA may be encouraged by these findings, continued monitoring of parasite clearance times is necessary to mitigate the adverse effects of developing resistance to artesunate.

## Supplementary Information

**Additional file 1: Table S1**. Individual counts used to generate rates over time in Figure 3. The top table shows numerators and the bottom table shows denominators. Note that the denominators vary over time to account for attrition, censoring, and varying follow-up across subjects.

## Data Availability

The datasets used and/or analysed during the current study are available from the corresponding author upon reasonable request.
