# Supplementary material for: Plasmodium falciparum clearance time in Malawian children with cerebral malaria: a retrospective cohort study
Source: Malar J. 2021 Oct 18;20:408. doi: 10.1186/s12936-021-03947-0 (PMC8524966; doi:10.1186/s12936-021-03947-0)
Supplement: Supplementary file 1 — Additional file 1: Table S1. Individual counts used to generate rates over time in Figure 3. The top table shows numerators and the bottom table shows denominators. Note that the denominators vary over time to account for attrition, censoring, and varying follow-up across subjects. [file 12936_2021_3947_MOESM1_ESM.docx]

Supplemental Table 1.

Individual counts used to generate rates over time in Figure 3. The top table shows numerators and the bottom table shows denominators. Note that the denominators vary over time to account for attrition, censoring, and varying follow-up across subjects.

**Cumulative count of those subjects reaching parasite count < 1000**

|  | 0 hrs | 6 hrs | 12 hrs | 18 hrs | 24 hrs | 30 hrs | 36 hrs | 42 hrs | 48 hrs |
| --- | --- | --- | --- | --- | --- | --- | --- | --- | --- |
| 2014 | 0 | 13 | 20 | 30 | 42 | 46 | 49 | 50 | 50 |
| 2015 | 0 | 7 | 11 | 17 | 24 | 28 | 28 | 28 | 28 |
| 2016 | 0 | 10 | 21 | 22 | 25 | 29 | 30 | 30 | 30 |
| 2017 | 0 | 12 | 19 | 23 | 32 | 39 | 40 | 40 | 40 |
| 2018 | 0 | 7 | 10 | 17 | 20 | 23 | 25 | 26 | 27 |
| 2019 | 0 | 5 | 9 | 12 | 13 | 14 | 17 | 18 | 18 |

**Count of those subjects who survived and parasite count > 1000**

|  | 0 hrs | 6 hrs | 12 hrs | 18 hrs | 24 hrs | 30 hrs | 36 hrs | 42 hrs | 48 hrs |
| --- | --- | --- | --- | --- | --- | --- | --- | --- | --- |
| 2014 | 53 | 53 | 39 | 32 | 20 | 8 | 4 | 1 | 0 |
| 2015 | 30 | 30 | 22 | 17 | 11 | 4 | 0 | 0 | 0 |
| 2016 | 30 | 30 | 20 | 9 | 8 | 5 | 1 | 0 | 0 |
| 2017 | 45 | 41 | 29 | 21 | 17 | 8 | 1 | 0 | 0 |
| 2018 | 29 | 28 | 20 | 17 | 10 | 7 | 4 | 2 | 1 |
| 2019 | 19 | 18 | 13 | 9 | 6 | 5 | 4 | 1 | 0 |
